# TRPV1 Contributes to Cerebral Malaria Severity and Mortality by Regulating Brain Inflammation

**DOI:** 10.1155/2019/9451671

**Published:** 2019-05-16

**Authors:** Domingos Magno Santos Pereira, Simone Aparecida Teixeira, Oscar Murillo, Erika Paula Machado Peixoto, Mizael Calácio Araújo, Nágila Caroline Fialho Sousa, Valério Monteiro-Neto, João Batista Calixto, Thiago Mattar Cunha, Cláudio Romero Farias Marinho, Marcelo Nicolás Muscará, Elizabeth Soares Fernandes

**Affiliations:** ^1^Programa de Pós-Graduação, Universidade CEUMA, São Luís, MA, Brazil; ^2^Departamento de Farmacologia, Instituto de Ciências Biomédicas, Universidade de São Paulo, São Paulo, SP, Brazil; ^3^Departamento de Parasitologia, Instituto de Ciências Biomédicas, Universidade de São Paulo, São Paulo, SP, Brazil; ^4^Centro de Ciências da Saúde, Universidade Federal do Maranhão, São Luís, MA, Brazil; ^5^Centro de Inovação e Ensaios Pré-Clínicos (CIEnP), Florianópolis, SC, Brazil; ^6^Departamento de Farmacologia, Faculdade de Medicina de Ribeirão Preto, Universidade de São Paulo, São Paulo, SP, Brazil

## Abstract

Transient receptor potential vanilloid 1 (TRPV1) is a Ca^+2^-permeable channel expressed on neuronal and nonneuronal cells, known as an oxidative stress sensor. It plays a protective role in bacterial infection, and recent findings indicate that this receptor modulates monocyte populations in mice with malaria; however, its role in cerebral malaria progression and outcome is unclear. By using TRPV1 wild-type (WT) and knockout (KO) mice, the importance of TRPV1 to this cerebral syndrome was investigated. Infection with *Plasmodium berghei* ANKA decreased TRPV1 expression in the brain. Mice lacking TRPV1 were protected against *Plasmodium*-induced mortality and morbidity, a response that was associated with less cerebral swelling, modulation of the brain expression of endothelial tight-junction markers (junctional adhesion molecule A and claudin-5), increased oxidative stress (via inhibition of catalase activity and increased levels of H_2_O_2_, nitrotyrosine, and carbonyl residues), and diminished production of cytokines. *Plasmodium* load was not significantly affected by TRPV1 ablation. Repeated subcutaneous administration of the selective TRPV1 antagonist SB366791 after malaria induction increased TRPV1 expression in the brain tissue and enhanced mouse survival. These data indicate that TRPV1 channels contribute to the development and outcome of cerebral malaria.

## 1. Introduction

Malaria is an infectious disease of great morbidity and mortality, which claimed the lives of more than 400 thousand people worldwide in 2015 [1]. Cerebral malaria is a clinical syndrome of the severe form of the disease and is characterized by neurological complications (coma and convulsions) associated with brain inflammation (for review, see [2]) which can be lethal or cause irreversible neurological and/or cognitive sequelae in surviving patients (for review, see [3]).

Several mechanisms were found to contribute to cerebral malaria including alterations in nitric oxide availability, unbalanced oxidative stress responses, changes in the pattern of expression of inflammatory molecules, vascular leakage, and blood brain barrier disruption, amongst others [4–10]. However, its treatment has proven to be difficult and of low efficacy depending on timing and parasite resistance [3, 11], with nearly 50% of the infected patients presenting this syndrome [3]. Importantly, 10-40% of the children with cerebral malaria die and a significant percentage develop sequelae [3, 12, 13]. In this context, the host response to infection plays a decisive role in the clinical evolution of malaria and therefore influences disease outcome.

The transient receptor potential vanilloid 1 (TRPV1) is a Ca^+2^-permeable channel expressed on neuronal and nonneuronal cells such as brain endothelial and immune cells [14–18], which plays a role in the inflammatory response of different pathologies (for review, see [16, 19]) and an emerging role in neuroinflammation (for review, see [20]). It was found that TRPV1 is protective against bacterial infection [21–24] and modulates the innate immune response to malaria [25]. These studies also indicate that TRPV1 is detrimental to macrophage/monocyte-mediated responses, including their ability to produce inflammatory mediators, especially those related to oxidative stress [23, 26–30], in addition to regulating body temperature [21, 23]. However, the relevance of TRPV1 to the brain inflammation and symptoms of cerebral malaria has never been investigated.

Here, we used TRPV1 wild-type (WT) and knockout (KO) mice to evaluate the role of TRPV1 in cerebral malaria. Disease progression and brain inflammation were assessed in mice infected with *Plasmodium berghei* ANKA. It was found that TRPV1 contributes to disease severity and mortality, by mediating brain inflammation.

## 2. Methods

### 2.1. Mice

Nonfasted male C57BL/6 wild-type (WT) and TRPV1 knockout (TRPV1KO) mice (2-3 months of age; 22-28 g) were used. Animals were obtained from the animal's facility of the Faculdade de Medicina de Ribeirão Preto, Universidade de São Paulo (USP). Mice (*n* = 3-4/cage) were housed in a climatically controlled environment (room temperature of 22 ± 2°C) and humidity of around 60%, on a 12-12 h light/dark cycle (lights on at 07:00), with free access to water and food. All experiments were conducted in accordance with the Brazilian Society for Animal Welfare (SBCAL), following approval by the Ethics Committee of USP. Animals were randomly assigned into groups, and the experimenter was blinded towards the genetic background of animals during the experiment. In some cases, C57BL/6 mice received the selective TRPV1 antagonist SB366791 (0.5 mg/kg, twice a day; Sigma-Aldrich, Brazil) or vehicle (10% DMSO in saline) for up to 14 days, starting at 24 h postmalaria induction. All assays were conducted in a blinded manner.

A total of 16 infected TRPV1 WT and 14 KO mice were used for analysis of survival rates, disease stage, and severity score; these data were obtained from two independent experiments. For performing the different biochemical, qPCR, and cytokine measurement experiments, samples were collected from 12 TRPV1 WT (5 noninfected and 07 infected) and 16 TRPV1 KO (5 noninfected and 11 infected) mice with stage III/IV malaria, in three independent experiments.

In two separate experiments, 20 WTs were used for assessment of mortality rates with the TRPV1 antagonist (10 vehicle-treated and 10 SB366791-treated). Disease stage and severity score experiments included 17 WT mice (8 vehicle-treated and 9 SB366791-treated), in two independent experiments. For experiments in animals with stage III/IV malaria, 11 TRPV1 WTs were used (5 vehicle-treated and 6 SB366791-treated) in two independent experiments.

### 2.2. Induction of Cerebral Malaria

Malaria was induced by a single intraperitoneal (i.p.) injection of 10^6^ red blood cells infected with *P. berghei* ANKA^GFP/HSP7^ as previously described [31, 32]. Parasitaemia and disease progression were evaluated from day 1 postinfection, by daily recording of parasitaemia and clinical neurological signs of cerebral malaria.

### 2.3. Blood Parasitaemia

The percentage of parasitaemia was determined by flow cytometry. For this, a drop of blood from the tail was collected directly into 2 ml of PBS for flow cytometry analysis. Each sample was run on a FACSCalibur (Becton Dickinson, San Jose, CA, USA) flow cytometer with a 488 nm argon laser and BD CellQuest™ Pro software version 6.0.1 (Becton Dickinson, San Jose, CA, USA). Erythrocytes were identified on the basis of their specific forward (FSC) and side (SSC) light-scattering properties, and a total of 100,000 events were counted for each sample.

### 2.4. Analysis of the Clinical Neurological Signs of Cerebral Malaria and Mortality Rates

The neurological signs were evaluated as described by Linares et al. [33], in order to determine disease progression (stages I-IV) as follows: stage I—presence of parasitaemia and absence of neurological symptoms; stage II—presence of head deviation or hemi- or paraplegia, in the absence or presence of piloerection, altered gait or ambulation, muscle weakness, tremor, rollover response, and/or anaemia; stage III—presence of significant neurological symptoms, including head deviation, paraplegia/hemiparaplegia, immobility, muscle weakness, piloerection, anaemia, pelvic elevation, lack of responses to external stimuli, tremor, and swollen eyes; and stage IV—presence of exacerbated neurological symptoms in comparison to those observed at stage III.

Disease severity was analysed by using the Rapid Murine Coma and Behavior Scale as previously described by Carroll et al. [34], with minor modifications. Briefly, a score from 0 (normal) to 2 (severe alteration) was attributed to each one of the following parameters as follows: (i) coordination (gait and balance), (ii) exploratory behavior (motor performance), (iii) strength and tone (body position and limb strength), (iv) reflexes and self-preservation (touch escape, pinna reflex, toe pinch, and aggression), and (v) hygiene-related behavior (grooming). The summation of the scores attributed to each of the parameters for each animal was taken as severity score index, with the highest scores corresponding to the worst outcome of disease.

The animals were observed for up to 14 days postinfection and were culled by anaesthetic overdose (90 mg/kg ketamine + 2 mg/kg xylazine; i.p.) as soon as they reached stage III/IV (premortality end point). Blood samples were collected, and the plasma was obtained. Brain samples were also collected and weighted. Collected plasma and brain tissue samples were immediately frozen and stored at -80°C until further processing for analysis of different parameters, except those used for qRT-PCR to which RNAlater was added according with the manufacturer's instructions (Sigma-Aldrich, Brazil).

Also, body weight and temperature were registered before (baseline) and at stage III/IV postinfection. All those which did not reach stage III/IV during the observation period were culled, and their measurements and samples were collected at the 14^th^ day postinfection. Noninfected mice were used as controls.

In a separate series of experiments, mortality rates were evaluated over 14 days following induction of cerebral malaria, in independent groups of mice.

### 2.5. Brain Parasite Load

Tissue parasite load was evaluated in brain samples (left hemisphere) collected from infected TRPV1 WT and KO mice, as previously described [33], and modified. Tissue parasite loads were determined by quantitative PCR and expressed as copy numbers of *P. berghei* ANKA 18S DNA per milligram of host tissue. For this, RNA was extracted in RNeasy Microarray Tissue Mini Kit, according to the manufacturer's instructions (Qiagen, Brazil). Then, the cDNA was prepared by reverse transcription of 2 *μ*g of RNA with ImProm-II Reverse Transcriptase (Promega, USA). The cDNA was assayed by qRT-PCR using the TaqMan® system (Applied Biosystems, USA) with *P. berghei* probes (AI 38261, PN 4332079). GAPDH levels were assessed by TaqMan Mouse GAPDH System (TaqMan®, Applied Biosystems, USA) and were used as housekeeping gene controls.

### 2.6. TRPV1, Junctional Adhesion Molecule-A (JAM-A), and Claudin-5 Gene Expression by Real-Time qPCR

qRT-PCR was performed using GoTaq qPCR Master Mix (Promega, USA) and a Rotor-Gene 6000 real-time PCR machine (Corbett Life Science, Australia) in a final volume of 12 *μ*l (hold: 2 min at 95°C; cycling: 40 cycles: 15 s at 95°C and 30 s at 60°C; melt: 68-90°C). The following primers were used: TRPV1 (forward 5′-GCGACCATCCCTCAAGAGT-3′, reverse 5′-CTTGCGATGGCTGAAGTACA-3′; 109 bp; accession number NM_001001445.2), JAM-A (forward 5′-GGTCAGCATCCACCTCACTGT-3′, reverse 5′-AGGTCAGCACTGCCCTGTTC-3′; 94 bp; accession number NM_172647), claudin-5 (forward 5′-GTGCCGGTGTCACAGAAGTA-3′, reverse 5′-GTACTTGACCGGGAAGCTGA-3′; 147 bp; accession number NM_013805), and GAPDH (forward 5′-AAGGTCATCCCAGAGCTGAA-3′, reverse 5′-CTGCTTCACCACCTTCTTGA-3′; 138 bp; accession number NM_008084.2). For each gene in each sample, 2^-efficiency×Ct^ values were calculated and divided by the corresponding value of 2^-efficiency×Ct^ obtained for GAPDH. In order to normalize the data, all the individual results were divided by the average value obtained for the control group (noninfected WT mice). Efficiencies were of 0.47, 0.59, 0.68, and 0.8 for TRPV1, claudin-5, JAM-A, and GAPDH primers, respectively.

### 2.7. Cytokine Measurements

Brain samples (right hemisphere) were prepared, and the supernatant was obtained as previously described [35] and used in the assays. The tissue and plasma levels of TNF*α*, IFN*γ*, and IL-6 were evaluated by using mouse cytometric bead array (CBA) cytokine kits according to the manufacturer's instructions (BD Biosciences, Brazil). Data analysis was performed on a FACSCalibur flow cytometer (BD Biosciences Immunocytometry Systems). Results were calculated in FCAP Array Software version 3.0.1 (BD Biosciences, Brazil) and expressed as picograms of cytokine per mg of tissue protein (pg/mg of protein) or picograms per milliliter of plasma (pg/ml).

### 2.8. Tissue Sample Preparation for Biochemical Analysis of Oxidative Stress Pathways

Brain samples (100 mg; right hemisphere) were homogenized in 1000 *μ*l of 0.05 M NaPO_4_ (pH 7.4) containing ethylenediaminetetraacetic acid (EDTA, 1 mM) and centrifuged at 10,000g, for 10 min, at 4°C, and then the supernatant was collected and stored at -80°C for analysis of enzyme activities.

### 2.9. Superoxide Dismutase (SOD)

SOD activity was measured as described by Abreu et al. [36]. Briefly, 10 *μ*l of each sample was incubated with 260 *μ*l of sodium carbonate buffer (50 mM; pH 9.4 containing 3 mM EDTA), 10 *μ*l of 3 mM xanthine, 10 *μ*l of 153 mU/ml of 2,3-bis-(2-methoxy-4-nitro-5-sulfophenyl)-2H-tetrazolium-5-carboxanilide (XTT), and 10 *μ*l of 1.87 mU/ml xanthine oxidase. Then, 200 *μ*l of the mixture was added per well in 96-well plates and the absorbance was read at 470 nm for 20 min. Blank reactions were prepared for each sample by boiling them for 5 min in order to inactivate SOD. Results are expressed as milliunits (mU) of SOD/mg of protein. Enzyme activity was defined as the ability of one unit of SOD to dismutate 1 *μ*mol of O_2_^−^/min.

### 2.10. Catalase

Catalase activity was measured as previously described [36], by incubating 30 *μ*l of brain homogenates or plasma samples with 500 *μ*l of hydrogen peroxide (H_2_O_2_, 10 mmol/ l) for 20 min, at 25 °C. Reactions were stopped with 500 *μ*l of sodium azide (1 mmol/l), and the concentration of the remaining H_2_O_2_ was determined by the oxidation of *o*-dianisidine. For this, 20 *μ*l of each reaction was incubated with 200 *μ*l of phosphate buffer (5 mM; pH 6.0) containing 0.167 mg/ml *o*-dianisidine and 0.095 mg/ml horseradish peroxidase (HRP). The absorbance was immediately read at 460 nm (SpectraMax Plus 384, Molecular Devices Inc., Sunnyvale, EUA) for 10 min. The remaining reactions were incubated at 60°C for 2 h, in order to inactivate catalase, and used as controls. A standard curve of H_2_O_2_ (11.3-8820 *μ*M) was used for comparison. Results are expressed as international units (IU) of catalase per milligram (mg) of protein. One IU of catalase was defined as the amount of H_2_O_2_ (in *μ*mol) degraded per minute.

### 2.11. Glutathione Peroxidase (GPx) and Reductase (GR)

GR activity was assessed by measuring the consumption of nicotinamide adenine dinucleotide phosphate (NADPH) as a cofactor in the reduction of oxidized glutathione (GSSG) to reduced GSH [36]. For this, 10 *μ*l of the sample was incubated with 190 *μ*l of a solution containing 2 mg/ml GSSG and 0.4 mg/ml NADPH, at 37°C. Absorbances were then recorded for 30 min (incubation period), at 340 nm. The results are expressed as *μ*mol of NADPH per min normalized per mg of protein (*μ*mol of NADPH/min/mg of protein).

GPx activity was determined as previously [36]. For this, 30 *μ*l of sample per well (diluted 1:3) was incubated for 5 min at 37°C, with 145 *μ*l per well of 0.05 M phosphate buffer (pH 7.4) containing 0.1 M EDTA, 5 *μ*l of glutathione (GSH, 80 mM), and 5 *μ*l glutathione reductase (0.0096 U/*μ*l). After incubation, 5 *μ*l of 0.46 % *tert*-butyl hydroperoxide solution and 10 *μ*l of 1.2 mM NADPH were added to each well. Absorbances were monitored at 340 nm for 10 min. The results are expressed as *μ*mol of GSH/min/mg of protein.

### 2.12. Thioredoxin Reductase (TrxR)

TrxR activity was determined by incubating 20 *μ*l of the sample with 140 *μ*l of assay buffer (0.05 M phosphate buffer (pH 7.4) containing 0.1 M EDTA, 50 mM potassium chloride, and 0.2 mg/ml bovine serum albumin), 20 *μ*l of 2 mM NADPH, and 20 *μ*l of 5 mM 5,5′-dithiobis(2-nitrobenzoic acid) (DTNB), in the presence and absence of a TrxR inhibitor (sodium aurothiomalate; 20 *μ*M) [37]. Absorbances were read at 412 nm for 5 min. The results are expressed in IU of TrxR per mg of protein (IU/mg of protein). Enzyme activity was defined as the NADPH-dependent production of 2 *μ*mol of 2-nitro-5-thiobenzoate per min at 22°C.

### 2.13. Protein Nitrotyrosine and Carbonyl Levels

For analysis of protein nitrotyrosine and carbonyl levels, 2.5 *μ*g of each sample was assayed by slot blotting. The presence of proteins containing 3-nitrotyrosine residues was analysed in the samples as previously described [38]. After sample derivatization by addition of Laemmli buffer (0.125 M Trizma, pH 6.8; 4% SDS and 20% glycerol; 20 min at room temperature and boiling for 2 min), the membrane was incubated with mouse monoclonal anti-nitrotyrosine primary antibody (1 : 2,000; Merck Millipore Co., Germany) overnight at 18°C.

Carbonylated proteins were determined according to the method described by Robinson et al. [39]. After the derivatization reaction by addition of 2,4-dinitrophenylhydrazine (DNPH) solution (0.1 mg/ml in 2N HCl, 5 min), the membrane was incubated with anti-DNP primary antibody (1 : 25,000 in blocking buffer, Abcam, UK) overnight at 18°C.

Immunoreactive bands were detected by chemiluminescence, and their intensities were estimated by densitometric analysis (ChemiDoc Image Systems, Bio-Rad, USA). Results were normalized by the band intensity values obtained after staining with Ponceau red.

### 2.14. Plasma and Tissue Hydrogen Peroxide Measurements

The levels of H_2_O_2_ were measured in brain homogenates and plasma samples by using a H_2_O_2_/peroxidase assay kit (Amplex Red H_2_O_2_/peroxidase assay kit; Molecular Probes, Invitrogen, Brazil) according to the manufacturer's instructions. Results were obtained by comparison of each sample with a H_2_O_2_ (0–10 *μ*M) standard curve and are expressed as H_2_O_2_ levels in *μ*M (plasma) and in picomoles of H_2_O_2_ per mg of protein (brain samples).

### 2.15. Statistical Analysis

The results are presented as mean ± standard error (SE). The percentage of inhibition is reported as the mean for each individual experiment. For multiple statistical comparisons between groups, data were analysed by repeated-measures analysis of variance (ANOVA) or one-way ANOVA followed by the Bonferroni test with FDR correction. Paired and unpaired *t* tests were used when appropriate. Survival curves were analysed by the nonparametric Mantel-Cox test. All data were analysed in GraphPad Prism 5.0. *p* < 0.05 was considered significant. All *n* numbers are indicated on the graphs.

## 3. Results

### 3.1. *P. berghei* Infection Reduces TRPV1 mRNA Expression in the Mouse Brain, a Response Attenuated by TRPV1 Antagonism

**Figure 1:**
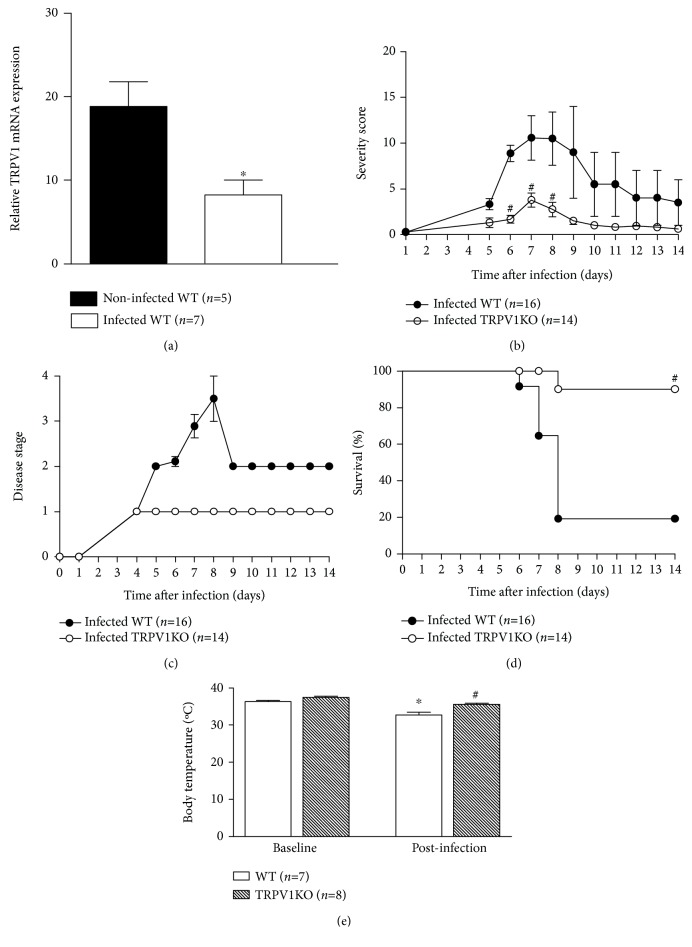
Brain TRPV1 mRNA expression and cerebral malaria progression. (a) TRPV1 mRNA expression in brain samples of noninfected and infected (at stage III/IV) TRPV1 wild-type (WT) mice. Disease progression (b) and stage (c); survival rates (d) and body temperature (e) recordings from TRPV1 WT and knockout (KO) mice infected with *P. berghei* ANKA. Disease progression, stage, and survival rates were registered over 14 days postinfection. Mouse body temperatures were evaluated at baseline and postmalaria induction (at stage III/IV or at day 14 for those that survived the observation period). Results represent the mean  ±  SEM of all mice per group, obtained from two-three independent experiments. *n* is indicated on each graph. Data were analysed by repeated-measures analysis of variance (ANOVA) followed by the Bonferroni test with FDR correction (panels b and c). Paired and unpaired *t* tests were used when appropriate (panels a and e). Survival curves were analysed by the nonparametric Mantel-Cox test (panel d). ^∗^ *p* < 0.05 differs from noninfected WTs or baseline readings; ^#^ *p* < 0.05 differs from infected WT mice.

We initially investigated whether infection with *P. berghei* ANKA, a plasmodium strain known to cause cerebral malaria in mice, influences TRPV1 expression in the mouse brain. Infected WT mice expressed lower TRPV1 mRNA levels (56%) in their brain tissue than noninfected controls did (Figure 1(a)). On the other hand, the systemic administration of SB366791 in C57BL/6 mice with malaria increased TRPV1 expression (2.1-fold increase) in comparison with vehicle controls (Supplementary Material Figure S1a).

### 3.2. Loss of TRPV1 Signaling Protects against Cerebral Malaria

We next assessed whether the ablation of TRPV1 influences cerebral malaria progression and mortality. Data depicted in Figure 1(b) show that infected TRPV1KO mice exhibit attenuated disease in comparison with WT controls. Of note, TRPV1KOs only presented parasitaemia without any other sign or symptom of cerebral malaria, suggesting they do not develop this syndrome. Accordingly, malaria was less severe and remained at stage I in these mice whilst it progressed into stages III and IV in the majority of the WT animals over the 14-day observation period (Figures 1(b) and 1(c)). Mortality was markedly prevented by TRPV1 ablation as 90% of the TRPV1KO mice survived infection in contrast with WT animals (19% survival; Figure 1(d)). Mice treated with SB366791 presented similar disease severity and course to those receiving vehicle until day 6 postinfection, improving their condition over the 14-day observation period (Supplementary Material Figures S1b and S1c). Twenty percent of those receiving the TRPV1 antagonist survived (Supplementary Material Figure S1d). As lack of TRPV1 was previously shown to exacerbate hypothermia in mice with bacterial infection [23], mouse body temperatures were registered. At baseline conditions, both genotypes exhibited similar body temperatures; however, hypothermia was only observed in stage III/IV WT but not TRPV1KO mice (Figure 1(e)). A similar response was registered in those receiving SB366791 (Supplementary Material Figure S1e).

**Figure 2:**
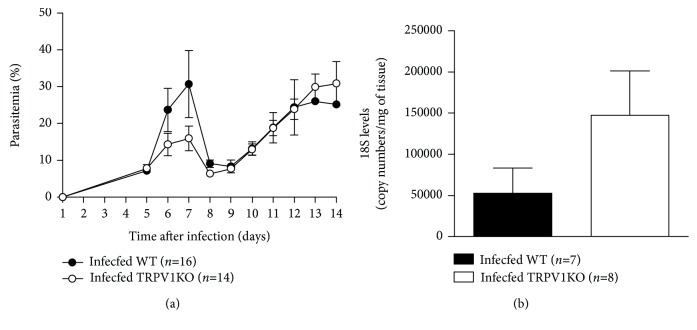
Blood and brain parasitaemia. (a) Blood parasitaemia and (b) brain 18S levels in TRPV1 wild-type (WT) and knockout (KO) mice infected with *P. berghei* ANKA. Blood parasitaemia data was collected over 14 days postinfection; brain samples were collected at stage III/IV or at day 14 for those that survived the observation period. Results represent the mean  ±  SEM of all mice per group, obtained from two-three independent experiments. *n* is indicated on each graph. Data were analysed by repeated-measures analysis of variance (ANOVA) followed by the Bonferroni test with FDR correction (panel a). Unpaired *t* test was used when appropriate (panel b).

Blood parasitaemia was similar in both genotypes, although WT mice exhibited higher parasitaemia than TRPV1KOs did at days 6 and 7 postinfection (Figure 2(a)). On the other hand, *P. berghei* ANKA 18S levels were elevated in the brain samples of TRPV1KO (2.8-fold) in comparison with those obtained from WT mice (Figure 2(b)).

### 3.3. Lack of TRPV1 Increases the mRNA Expression of Blood Brain Barrier Integrity Markers and Attenuates Oedema Formation in the Brains of Infected Mice

**Figure 3:**
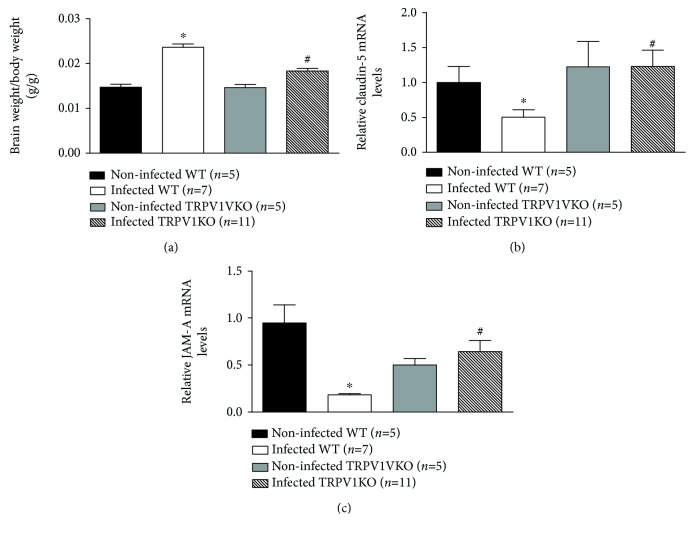
Brain swelling and expression of blood brain barrier integrity markers. (a) Brain weight/body weight ratios and mRNA expression levels of claudin-5 (b) and JAM-A (c) in brain samples of TRPV1 wild-type (WT) and knockout (KO) mice infected with *P. berghei* ANKA. Brain samples were collected at stage III/IV or at day 14 for those that survived the observation period. Samples from noninfected mice were used as controls. Results represent the mean  ±  SEM of all mice per group, obtained from three independent experiments. *n* is indicated on each graph. Data were analysed by one-way analysis of variance (ANOVA) followed by the Bonferroni test with FDR correction. ^∗^ *p* < 0.05 differs from noninfected WTs; ^#^ *p* < 0.05 differs from infected WT mice.

Loss of integrity of the blood brain barrier is a hallmark of cerebral malaria, contributing to increased oedema formation and neuronal damage as disease progresses [40, 41]. Possible effects of TRPV1 ablation in brain oedema formation and in the gene expression of the tight junction components claudin-5 and JAM-A [41, 42] were then, investigated. Data depicted in Figure 3(a) demonstrates that infection with *P. berghei* ANKA promotes brain swelling in WT (1.7-fold) and TRPV1KO (1.2-fold) mice in comparison with their respective noninfected controls; however, this response was reduced by 25% in those lacking TRPV1. Additionally, analysis of claudin-5 and JAM-A mRNA levels revealed that infected WT mice express diminished levels of both genes (49% and 80%, respectively), in comparison with noninfected controls, a response that was attenuated in infected TRPV1KO mice (Figures 3(b) and 3(c)). Genotype did not affect brain weight/body weight ratios or claudin-5 mRNA expression in noninfected mice (Figures 3(a) and 3(b)). However, noninfected TRPV1KOs presented with lower expression of JAM-A (47%) in comparison with their WT counterparts (Figure 3(c)).

### 3.4. H_2_O_2_, Protein Nitrotyrosine and Carbonyl Residues Are Raised in Infected TRPV1KO Animals

**Figure 4:**
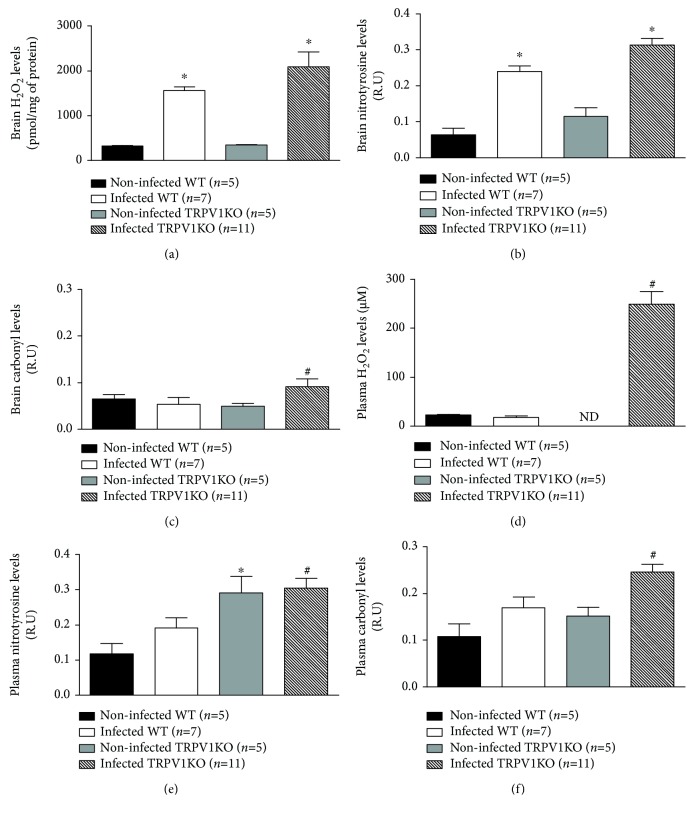
Levels of H_2_O_2_, protein nitrotyrosine, and carbonyl residues. (a) H_2_O_2_ concentrations, protein (b) nitrotyrosine and (c) carbonyl residues in brain samples obtained from TRPV1 wild-type (WT) and knockout (KO) mice infected or not with *P. berghei* ANKA. (d) H_2_O_2_ concentrations, protein (e) nitrotyrosine, and (f) carbonyl residues in plasma samples obtained from TRPV1 wild-type (WT) and knockout (KO) mice infected or not with *P. berghei* ANKA. Samples were collected at stage III/IV or at day 14 for those that survived the observation period. Results represent the mean  ±  SEM of all mice per group, obtained from three independent experiments. *n* is indicated on each graph. Data were analysed by one-way analysis of variance (ANOVA) followed by the Bonferroni test with FDR correction. ^∗^ *p* < 0.05 differs from noninfected WTs; ^#^ *p* < 0.05 differs from infected WT mice.

Oxidative stress normally occurs as part of the host response to malaria [5, 43]. TRPV1 is an oxidative stress sensor [28], which not only does modulate oxidative stress [23, 27] but also can have its expression regulated by endogenous oxidant molecules [26]. Therefore, the impact of TRPV1 ablation in malaria-associated oxidative stress was investigated. Higher levels of H_2_O_2_ and protein nitrotyrosine residues (indicative of NO-dependent oxidative stress; [44]) were detected in infected mice of both genotypes in comparison with their noninfected controls (Figures 4(a) and 4(b)). WT mice presented 4.8-fold and 3.7-fold increases and TRPV1KOs 6.0-fold and 2.7-fold increases for tissue H_2_O_2_ and protein nitrotyrosine residue levels, respectively. Protein carbonyl residues (indicative of lipid peroxidation-dependent oxidative stress; [45]) were only increased (1.9-fold) in brain samples of infected mice lacking TRPV1 (Figure 4(c)). Analysis of plasma H_2_O_2_ levels, and protein nitrotyrosine and carbonyl levels indicated these were raised in TRPV1KO but not WT mice infected with *P. berghei* ANKA (Figures 4(d)–4(f)). TRPV1KOs presented greater levels of plasma H_2_O_2_ (13.9-fold increase), protein nitrotyrosine (1.5-fold increase), and carbonyl (1.4-fold increase) residues in comparison with those observed for WT animals with cerebral malaria (Figures 4(d)–4(f)).

**Figure 5:**
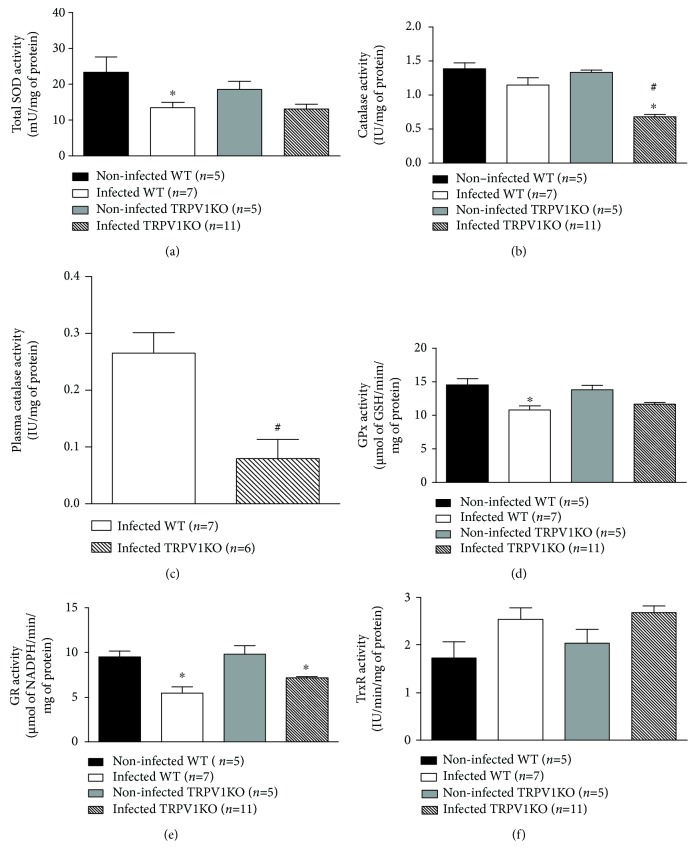
Activity levels of antioxidant enzymes. (a) Superoxide dismutase (SOD), (b) catalase, (d) glutathione peroxidase (GPx), (e) glutathione reductase (GR), and (f) thioredoxin reductase (TrxR) activity levels in brain samples obtained from TRPV1 wild-type (WT) and knockout (KO) mice infected or not with *P. berghei* ANKA. (c) Activity levels of catalase in plasma samples of infected TRPV1 WT and KO mice. Samples were collected at stage III/IV or at day 14 for those that survived the observation period. Results represent the mean  ±  SEM of all mice per group, obtained from three independent experiments. *n* is indicated on each graph. Data were analysed by one-way analysis of variance (ANOVA) followed by the Bonferroni test with FDR correction (panels a, b, d, e, and f). Unpaired *t* test was used when appropriate (panel c). ^∗^ *p* < 0.05 differs from noninfected WTs; ^#^ *p* < 0.05 differs from infected WT mice.

As TRPV1KO mice presented with an exacerbated production of oxidants, the activity of antioxidant enzymes was then, investigated. The tissue activity levels of SOD, GPx, and GR were attenuated (by ~35%, ~20%, and ~34% of reduction, respectively) in infected mice irrespective of genotype when compared to noninfected controls (Figures 5(a), 5(d), and 5(e)). Also, TrxR activity was enhanced in both infected genotypes (1.5-fold increase; Figure 5(f)). On the other hand, brain catalase activity was markedly diminished (49%) in infected TRPV1KO but not WT mice (Figure 5(b)). Infected TRPV1KO mice also displayed lower levels of catalase activity (70% less) in their plasma in comparison with WT controls (Figure 5(c)).

### 3.5. Diminished Cytokine Production Is Detected in Infected TRPV1KO Mice

**Figure 6:**
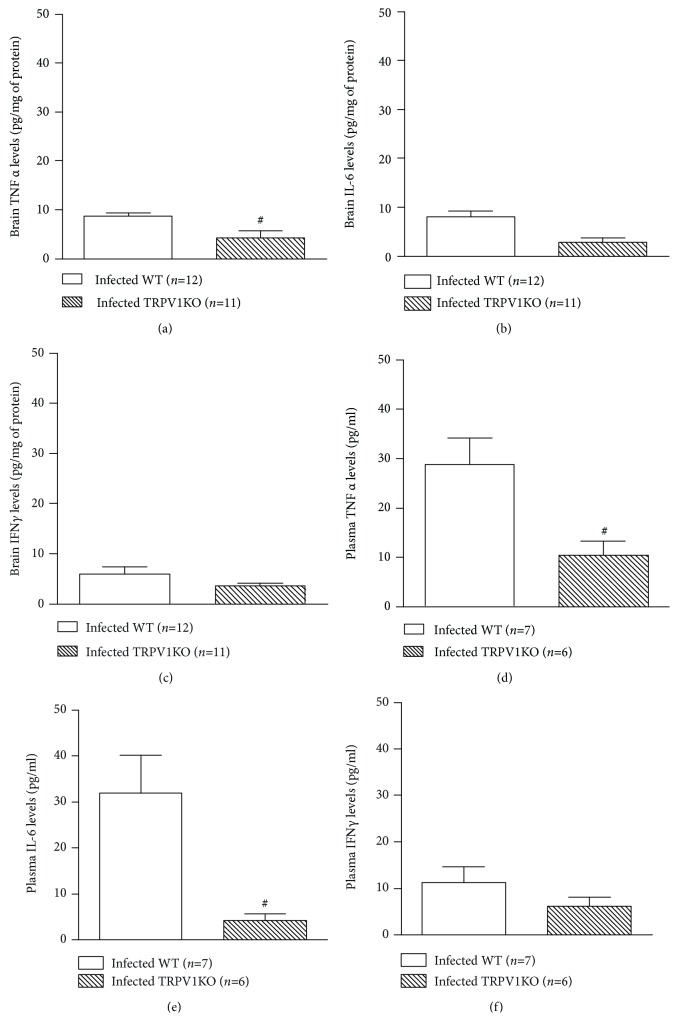
Brain and circulating levels of cytokines. Brain levels of (a) tumor necrosis *α* (TNF*α*), (b) interleukin-6 (IL-6), and (c) interferon *γ* (IFN*γ*) and plasma concentrations of (d) TNF*α*, (e) IL-6, and IFN*γ* (f) in TRPV1 wild-type (WT) and knockout (KO) mice infected with *P. berghei* ANKA. Samples were collected at stage III/IV or at day 14 for those that survived the observation period. Results represent the mean  ±  SEM of all mice per group, obtained from three independent experiments. *n* is indicated on each graph. Data were analysed by unpaired *t* test. ^#^ *p* < 0.05 differs from infected WT mice.

Cytokines are involved in neuronal survival [46, 47] and therefore may affect cerebral malaria progression. Thus, the levels of both tissue and plasma IFN*γ*, TNF*α*, and IL-6 were assessed in WT and TRPV1KO mice with malaria. Tissue and plasma TNF*α* production was markedly reduced (52% and 64%, respectively; Figures 6(a) and 6(d)) in TRPV1KO in comparison with WT controls. A similar profile was observed for IL-6 as mice lacking TRPV1 exhibited significant lower levels of this cytokine at both tissue (65% reduction) and plasma (86% reduction) levels (Figures 6(b) and 6(d)). Genotype did not affect IFN*γ* levels in a significant manner (Figures 6(c) and 6(f)).

## 4. Discussion

Since its discovery, the TRPV1 channel has been pointed out as an essential receptor in a variety of physiological and pathological responses. This is due to its wide expression and ability to transduce signals in both neuronal and nonneuronal cells, therefore participating in responses that range from cell differentiation to death [16, 23, 48–50]. Novel and recent findings on its role indicate that the endogenous activation of TRPV1 protects mammals from bacterial infections [21–24]. More recently, a nonselective TRPV1 antagonist (capsazepine) was found to modulate the peripheral immune response to malaria [25], but no studies have reported to date, a role for TRPV1 in cerebral malaria development and outcome. Here, we show for the first time that in the absence of TRPV1, *P. berghei* ANKA infection does not progress into cerebral malaria in the majority of the infected mice, protecting them from death and from the development of any disease symptoms and signals apart from blood parasitaemia. Protection was also observed in mice receiving the TRPV1 antagonist SB366791 repeatedly after malaria was induced. Of note, this effect was more pronounced in TRPV1KOs than in mice treated with SB366791. Although these results suggest that an intervention with a TRPV1 antagonist may be an alternative to avoid malaria progression, its use should be carefully considered as it may increase mortality upon bacterial infection [23]. Interestingly, although TRPV1 ablation exacerbates hypothermia in bacterial infection [21, 23], it was found herein that TRPV1KO mice and WTs treated with the selective TRPV1 antagonist SB366791 are protected from this condition in comparison with infected WTs.

*P. berghei* ANKA-infected mice treated with capsazepine were previously demonstrated to present similar blood parasitaemia to those treated with vehicle [25]. Here, we show that infected mice lacking TRPV1 present with similar blood parasitaemia to those expressing this receptor. On the other hand, at days 6 and 7 postinfection, infected WTs presented higher parasitaemia than TRPV1KOs did. Despite that, surviving TRPV1KO mice exhibited higher levels of plasmodium 18S in their brain samples than WTs did with cerebral malaria at stage III/IV. Of note, the techniques used to measure blood parasitaemia and brain parasite load are different as peripheral parasitaemia comprises the detection of live parasites whilst brain 18S expression does not discriminate between live and dead plasmodium. However, it is possible that TRPV1KO mice are able to kill the parasites that reach the brain more efficiently than WTs are, therefore protecting those lacking TRPV1 from death.

Brain oedema formation in patients with cerebral malaria is indicative of a bad disease prognosis, especially in children [3, 51]. In adults, brain oedema is not as usual but affects 25% of these patients [52]. Brain swelling results from increased vascular leakage and disruption of the blood brain barrier [8, 9]. TRPV1 activation promotes vasodilation and oedema formation [53, 54]. Then, the contribution of TRPV1 to brain oedema formation was assessed in infected mice. Infected WT mice exhibited brain swelling and decreased mRNA expression of the markers of blood brain barrier integrity JAM-A and claudin-5 [41, 42]. However, in the absence of TRPV1, there was higher JAM-A and claudin-5 mRNA expression. This response was associated with less brain oedema formation, suggesting that mice lacking TRPV1 are protected from the brain damage, coma, and death associated with protein leakage into the brain tissue secondary to plasmodium infection.

Intravascular oxidative stress is a common phenomenon in malaria which has been associated with alterations in the endothelium that in turn, facilitate the parasite accumulation into the brain tissue and/or vasculature [55, 56]. Additionally, decreased NO availability was recently linked to increased cerebral-vascular dysfunction in cerebral malaria [6]. A feedback between TRPV1 expression/activation and oxidative stress pathways has been previously demonstrated [23, 26–28]. Of note, the activity of oxidative stress enzymes has been investigated in neurons under inflammatory conditions and may influence neuronal survival [57–61]. Therefore, the influence of TRPV1 on brain oxidative stress was evaluated.

Our data show that infected WTs present higher levels of H_2_O_2_ and protein nitrotyrosine residues (indicative of excessive NO- or peroxynitrite-dependent oxidation; [44]) in their brain tissue than noninfected mice do. Interestingly, these markers were present at even higher concentrations in mice lacking TRPV1. Of note, the elevated production of these oxidant products was observed not only in the brain tissue but also systemically. In comparison with infected WTs, TRPV1KO mice injected with *P. berghei* also exhibited increased protein carbonylation (indicative of lipid peroxidation-dependent oxidative stress; [45]) in brain and plasma samples. The higher levels of H_2_O_2_ in infected TRPV1KO were accompanied by a significantly lower catalase activity in comparison with WT animals. These results reinforce the idea that TRPV1KO mice may be able to deal with the parasite load more efficiently than WTs. This is supported by data showing that TRPV1KO mice present higher H_2_O_2_ and NO production which may lead to increased parasite killing.

High levels of cytokines have been linked to severe malaria in both humans and mice as their production contributes to cerebral-vascular dysfunction and even neuronal death [46, 47, 62–65]. Of note, lipid peroxidation is suggested to cause suppression of NF-*κ*B activation (for review, see [66]), a key molecule in the generation of proinflammatory cytokines. Here, plasma and cerebral TNF*α* and IL-6 production was markedly diminished by TRPV1 ablation, thus evidencing, once more, that TRPV1 signaling is involved in the tissue damage associated with cerebral malaria. Although not significant, a similar profile was observed for IFN*γ* in the same mice. Interestingly, IFN*γ* and TNF*α* have been linked to cerebral malaria progression by acting on brain endothelial cells, thus promoting their activation and/or apoptosis [67, 68]. Recently, the TRPV1 antagonist AMG9810 was found to confer neuroprotection by attenuating TNF*α* production in a rodent model of stroke [54]. These evidences and the gathered data allow us to suggest that the diminished cytokine generation by TRPV1KO mice contributes to the diminished brain swelling and damage observed in *P. berghei* ANKA-infected mice, a response that is associated with a greater ability of these mice to produce higher amounts of oxygen/nitrogen-derived oxidant species which in turn may enhance their capacity of killing this parasite.

**Figure 7:**
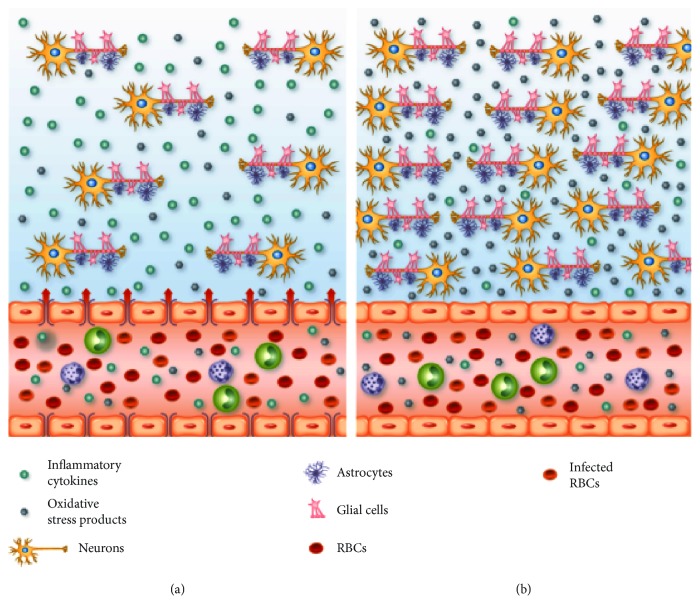
Brain and vascular changes in cerebral malaria in TRPV1 WT and KO mice. (a) Several alterations occur in the brain tissue and vasculature during cerebral malaria. Wild-type (WT) red blood cells (RBC) infected with *Plasmodium berghei* ANKA reach the brain vasculature and trigger the accumulation of leukocytes in the vascular space. As a result of this close interaction between infected RBC, leukocytes, and the endothelium, oxidative stress products (H_2_O_2_, nitrosylated and carbonylated proteins) and cytokines (TNF*α*, IL-6, and IFN*γ*) are detected in the circulation and in the brain tissue; H_2_O_2_ levels are a lot higher in the brain tissue in comparison with the circulation. Plasma extravazation is increased in the brain and this is associated with reduced mRNA expression of the tight-junction endothelial markers claudin-5 and JAM-A. These alterations may culminate with neuronal death, thus, contributing to the increased morbidity and mortality observed in WT mice following infection with *P. berghei* ANKA. (b) Infected mice lacking TRPV1 (TRPV1KO) present increased levels of H_2_O_2_ and nitrosylated and carbonylated proteins than WT animals at both brain tissue and circulation. TRPV1KOs also exhibit lower concentrations of plasma and brain cytokines, especially TNF*α* and IL-6, and less plasma extravazation than WT mice, a response that is accompanied by higher expression of claudin-5 and JAM-A in their brain vasculature. The inflammatory response profile observed in TRPV1KO mice may reflect in less neuronal damage, as these animals are protected from *P. berghei* ANKA-induced death and symptoms.

Figure 7 summarizes the inflammatory events that occur in the brain of TRPV1 WT and KO mice during cerebral malaria. Overall, the data presented here, indicate that TRPV1 channels contribute to the development and outcome of cerebral malaria. Although antagonists targeting this receptor may be useful to preventing the development of the cerebral syndrome caused by *Plasmodium* sp., their clinical use may be limited as they worsen sepsis outcome.

## Data Availability

The datasets used to support this study will be made available upon request. Requests should be sent to the corresponding author.

## Supplementary Materials

Supplementary Materials Figure S1: Effect of the selective TRPV1 antagonist SB366791 in the brain expression of TRPV1 mRNA and in cerebral malaria progression. (a) TRPV1 mRNA expression in brain samples of infected (at stage III/IV) TRPV1 wild-type (WT) mice. Disease progression (b) and stage (c); survival rates (d) and body temperature (e) recordings from TRPV1 WT mice infected with P. berghei ANKA. Disease progression, stage, and survival rates were registered over 14 days postinfection. Mouse body temperatures were evaluated at baseline and postmalaria induction (at stage III/IV or at day 14 for those that survived the observation period). Mice received the TRPV1 antagonist SB366791 (0.5 mg/kg, s.c., twice a day) or vehicle (10% DMSO in saline), from 24 h postinduction of malaria. Results represent the mean + SEM of all mice per group, obtained from three independent experiments. *n* is indicated on each graph. ^∗^ *p* < 0.05 differs from baseline readings; ^#^ *p* < 0.05 differs from infected WT mice treated with vehicle.

## References

[1] World Health Organization (2016). *World Malaria Report 2016*.

[2] Shikani H. J., Freeman B. D., Lisanti M. P., Weiss L. M., Tanowitz H. B., Desruisseaux M. S. (2012). Cerebral malaria: we have come a long way. *The American Journal of Pathology*.

[3] World Health Organization (2014). Severe Malaria. *Tropical Medicine & International Health*.

[4] Gramaglia I., Sobolewski P., Meays D. (2006). Low nitric oxide bioavailability contributes to the genesis of experimental cerebral malaria. *Nature Medicine*.

[5] Narsaria N., Mohanty C., Das B. K., Mishra S. P., Prasad R. (2012). Oxidative stress in children with severe malaria. *Journal of Tropical Pediatrics*.

[6] Ong P. K., Melchior B., Martins Y. C. (2013). Nitric oxide synthase dysfunction contributes to impaired cerebroarteriolar reactivity in experimental cerebral malaria. *PLoS Pathogens*.

[7] Hernandez-Valladares M., Rihet P., Iraqi F. A. (2014). Host susceptibility to malaria in human and mice: compatible approaches to identify potential resistant genes. *Physiological Genomics*.

[8] Hackett M. J., Aitken J. B., el-Assaad F. (2015). Mechanisms of murine cerebral malaria: multimodal imaging of altered cerebral metabolism and protein oxidation at hemorrhage sites. *Science Advances*.

[9] Dunst J., Kamena F., Matuschewski K. (2017). Cytokines and chemokines in cerebral malaria pathogenesis. *Frontiers in Cellular and Infection Microbiology*.

[10] Strangward P., Haley M. J., Shaw T. N. (2017). A quantitative brain map of experimental cerebral malaria pathology. *PLoS Pathogens*.

[11] Marks M., Gupta-Wright A., Doherty J. F., Singer M., Walker D. (2014). Managing malaria in the intensive care unit. *British Journal of Anaesthesia*.

[12] Dondorp A., Nosten F., Stepniewska K., Day N., White N. (2005). Artesunate versus quinine for treatment of severe falciparum malaria: a randomised trial. *The Lancet*.

[13] Dondorp A. M., Fanello C. I., Hendriksen I. C. (2010). Artesunate versus quinine in the treatment of severe falciparum malaria in African children (AQUAMAT): an open-label, randomised trial. *The Lancet*.

[14] Golech S. A., McCarron R. M., Chen Y. (2004). Human brain endothelium: coexpression and function of vanilloid and endocannabinoid receptors. *Molecular Brain Research*.

[15] Tóth A., Boczán J., Kedei N. (2005). Expression and distribution of vanilloid receptor 1 (TRPV1) in the adult rat brain. *Molecular Brain Research*.

[16] Fernandes E. S., Fernandes M. A., Keeble J. E. (2012). The functions of TRPA1 and TRPV1: moving away from sensory nerves. *British Journal of Pharmacology*.

[17] Martins D., Tavares I., Morgado C. (2014). “Hotheaded”: the role of TRPV1 in brain functions. *Neuropharmacology*.

[18] Assas B. M., Abdulaal W. H., Wakid M. H., Zakai H. A., Miyan J., Pennock J. L. (2017). The use of flow cytometry to examine calcium signalling by TRPV1 in mixed cell populations. *Analytical Biochemistry*.

[19] Vetter I., Kym P. R., Szallasi A. (2015). Feeling hot, feeling cold: TRP channels-a great story unfolds. *Temperature*.

[20] Kong W. L., Peng Y. Y., Peng B. W. (2017). Modulation of neuroinflammation: Role and therapeutic potential of TRPV1 in the neuro-immune axis. *Brain, Behavior, and Immunity*.

[21] Clark N., Keeble J., Fernandes E. S. (2007). The transient receptor potential vanilloid 1 (TRPV1) receptor protects against the onset of sepsis after endotoxin. *The FASEB Journal*.

[22] Guptill V., Cui X., Khaibullina A. (2011). Disruption of the transient receptor potential vanilloid 1 can affect survival, bacterial clearance, and cytokine gene expression during murine sepsis. *Anesthesiology*.

[23] Fernandes E. S., Liang L., Smillie S. J. (2012). TRPV1 deletion enhances local inflammation and accelerates the onset of systemic inflammatory response syndrome. *Journal of Immunology*.

[24] Wanner S. P., Garami A., Pakai E. (2012). Aging reverses the role of the transient receptor potential vanilloid-1 channel in systemic inflammation from anti-inflammatory to proinflammatory. *Cell Cycle*.

[25] Fernandes E. S., Brito C. X. L., Teixeira S. A. (2014). TRPV1 antagonism by capsazepine modulates innate immune response in mice infected with *Plasmodium berghei* ANKA. *Mediators of Inflammation*.

[26] Puntambekar P., Mukherjea D., Jajoo S., Ramkumar V. (2005). Essential role of Rac1/NADPH oxidase in nerve growth factor induction of TRPV1 expression. *Journal of Neurochemistry*.

[27] Starr A., Graepel R., Keeble J. (2008). A reactive oxygen species-mediated component in neurogenic vasodilatation. *Cardiovascular Research*.

[28] Keeble J. E., Bodkin J. V., Liang L. (2009). Hydrogen peroxide is a novel mediator of inflammatory hyperalgesia, acting via transient receptor potential vanilloid 1-dependent and independent mechanisms. *Pain*.

[29] Schilling T., Eder C. (2009). Importance of the non-selective cation channel TRPV1 for microglial reactive oxygen species generation. *Journal of Neuroimmunology*.

[30] Schilling T., Eder C. (2010). Stimulus-dependent requirement of ion channels for microglial NADPH oxidase-mediated production of reactive oxygen species. *Journal of Neuroimmunology*.

[31] Elias R. M., Correa-Costa M., Barreto C. R. (2012). Oxidative stress and modification of renal vascular permeability are associated with acute kidney injury during *P. berghei* ANKA infection. *PLoS One*.

[32] Miranda A. S., Brant F., Rocha N. P. (2013). Further evidence for an anti-inflammatory role of artesunate in experimental cerebral malaria. *Malaria Journal*.

[33] Linares M., Marín-García P., Pérez-Benavente S. (2013). Brain-derived neurotrophic factor and the course of experimental cerebral malaria. *Brain Research*.

[34] Carroll R. W., Wainwright M. S., Kim K. Y. (2010). A rapid murine coma and behavior scale for quantitative assessment of murine cerebral malaria. *PLoS One*.

[35] Fernandes E. S., Passos G. F., Campos M. M. (2005). Cytokines and neutrophils as important mediators of platelet‐activating factor‐induced kinin B_1_ receptor expression. *British Journal of Pharmacology*.

[36] Abreu F. F., Souza A. C. A., Teixeira S. A. (2016). Elucidating the role of oxidative stress in the therapeutic effect of rutin on experimental acute pancreatitis. *Free Radical Research*.

[37] Hill K. E., McCollum G. W., Burk R. F. (1997). Determination of thioredoxin reductase activity in rat liver supernatant. *Analytical Biochemistry*.

[38] Sultana R., Butterfield D. A. (2008). Slot-blot analysis of 3-nitrotyrosine-modified brain proteins. *Methods in Enzymology*.

[39] Robinson C. E., Keshavarzian A., Pasco D. S., Frommel T. O., Winship D. H., Holmes E. W. (1999). Determination of protein carbonyl groups by immunoblotting. *Analytical Biochemistry*.

[40] Rénia L., Howland S. W., Claser C. (2012). Cerebral malaria: mysteries at the blood-brain barrier. *Virulence*.

[41] Nacer A., Movila A., Sohet F. (2014). Experimental cerebral malaria pathogenesis–hemodynamics at the blood brain barrier. *PLoS Pathogens*.

[42] Stamatovic S. M., Sladojevic N., Keep R. F., Andjelkovic A. V. (2012). Relocalization of junctional adhesion molecule A during inflammatory stimulation of brain endothelial cells. *Molecular and Cellular Biology*.

[43] Percário S., Moreira D. R., Gomes B. A. (2012). Oxidative stress in malaria. *International Journal of Molecular Sciences*.

[44] Kissner R., Nauser T., Kurz C., Koppenol W. H. (2004). Peroxynitrous acid - where is the hydroxyl radical?. *IUBMB Life*.

[45] Suzuki Y. J., Carini M., Butterfield D. A. (2010). Protein carbonylation. *Antioxidants & Redox Signaling*.

[46] Wiese L., Hempel C., Penkowa M., Kirkby N., Kurtzhals J. A. L. (2008). Recombinant human erythropoietin increases survival and reduces neuronal apoptosis in a murine model of cerebral malaria. *Malaria Journal*.

[47] Rodney T., Osier N., Gill J. (2018). Pro- and anti-inflammatory biomarkers and traumatic brain injury outcomes: a review. *Cytokine*.

[48] Stock K., Garthe A., de Almeida Sassi F., Glass R., Wolf S. A., Kettenmann H. (2014). The capsaicin receptor TRPV1 as a novel modulator of neural precursor cell proliferation. *Stem Cells*.

[49] Ramírez-Barrantes R., Cordova C., Poblete H. (2016). Perspectives of TRPV1 function on the neurogenesis and neural plasticity. *Neural Plasticity*.

[50] Amantini C., Farfariello V., Cardinali C. (2017). The TRPV1 ion channel regulates thymocyte differentiation by modulating autophagy and proteasome activity. *Oncotarget*.

[51] Potchen M. J., Kampondeni S. D., Seydel K. B. (2012). Acute brain MRI findings in 120 Malawian children with cerebral malaria: new insights into an ancient disease. *American Journal of Neuroradiology*.

[52] Cordoliani Y. S., Sarrazin J. L., Felten D., Caumes E., Lévêque C., Fisch A. (1998). MR of cerebral malaria. *American Journal of Neuroradiology*.

[53] Keeble J., Russell F., Curtis B., Starr A., Pinter E., Brain S. D. (2005). Involvement of transient receptor potential vanilloid 1 in the vascular and hyperalgesic components of joint inflammation. *Arthritis and Rheumatism*.

[54] Hakimizadeh E., Shamsizadeh A., Roohbakhsh A. (2017). Inhibition of transient receptor potential vanilloid-1 confers neuroprotection, reduces tumor necrosis factor-alpha, and increases IL-10 in a rat stroke model. *Fundamental & Clinical Pharmacology*.

[55] Kumar S., Bandyopadhyay U. (2005). Free heme toxicity and its detoxification systems in human. *Toxicology Letters*.

[56] Phiri H., Montgomery J., Molyneux M., Craig A. (2009). Competitive endothelial adhesion between *Plasmodium falciparum* isolates under physiological flow conditions. *Malaria Journal*.

[57] Guo L. L., Guan Z. Z., Huang Y., Wang Y. L., Shi J. S. (2013). The neurotoxicity of *β*-amyloid peptide toward rat brain is associated with enhanced oxidative stress, inflammation and apoptosis, all of which can be attenuated by scutellarin. *Experimental and Toxicologic Pathology*.

[58] Wei J., Fang W., Sha L. (2013). XQ-1H suppresses neutrophils infiltration and oxidative stress induced by cerebral ischemia injury both in vivo and in vitro. *Neurochemical Research*.

[59] Jung H. Y., Kim D. W., Yim H. S. (2016). Heme oxygenase-1 protects neurons from ischemic damage by upregulating expression of Cu,Zn-superoxide dismutase, catalase, and brain-derived neurotrophic factor in the rabbit spinal cord. *Neurochemical Research*.

[60] Yang S. J., Kim E. A., Chang M. J. (2017). *N*-Adamantyl-4-methylthiazol-2-amine attenuates glutamate-induced oxidative stress and inflammation in the brain. *Neurotoxicity Research*.

[61] Cohen-Kutner M., Khomsky L., Trus M. (2014). Thioredoxin-mimetic peptide CB3 lowers MAPKinase activity in the Zucker rat brain. *Redox Biology*.

[62] Armah H. B., Wilson N. O., Sarfo B. Y. (2007). Cerebrospinal fluid and serum biomarkers of cerebral malaria mortality in Ghanaian children. *Malaria Journal*.

[63] John C. C., Panoskaltsis-Mortari A., Opoka R. O. (2008). Cerebrospinal fluid cytokine levels and cognitive impairment in cerebral malaria. *The American Journal of Tropical Medicine and Hygiene*.

[64] Krupka M., Seydel K., Feintuch C. M. (2012). Mild Plasmodium falciparum malaria following an episode of severe malaria is associated with induction of the interferon pathway in Malawian children. *Infection and Immunity*.

[65] Mandala W. L., Msefula C. L., Gondwe E. N., Drayson M. T., Molyneux M. E., MacLennan C. A. (2017). Cytokine profiles in Malawian children presenting with uncomplicated malaria, severe malarial anemia, and cerebral malaria. *Clinical and Vaccine Immunology*.

[66] Schwarzer E., Arese P., Skorokhod O. A. (2015). Role of the lipoperoxidation product 4-hydroxynonenal in the pathogenesis of severe malaria anemia and malaria immunodepression. *Oxidative Medicine and Cellular Longevity*.

[67] Hunt N. H., Ball H. J., Hansen A. M. (2014). Cerebral malaria: gamma-interferon redux. *Frontiers in Cellular and Infection Microbiology*.

[68] Villegas-Mendez A., Strangward P., Shaw T. N. (2017). Gamma interferon mediates experimental cerebral malaria by signaling within both the hematopoietic and nonhematopoietic compartments. *Infection and Immunity*.

