# Supplementary material for: TRPV1 Contributes to Cerebral Malaria Severity and Mortality by Regulating Brain Inflammation
Source: Oxid Med Cell Longev. 2019 May 16;2019:9451671. doi: 10.1155/2019/9451671 (PMC6541938; doi:10.1155/2019/9451671)
Supplement: Supplementary Materials — Figure S1: Effect of the selective TRPV1 antagonist SB366791 in the brain expression of TRPV1 mRNA and in cerebral malaria progression. (a) TRPV1 mRNA expression in brain samples of infected (at stage III/IV) TRPV1 wild-type (WT) mice. Disease progression (b) and stage (c); survival rates (d) and body temperature (e) recordings from TRPV1 WT mice infected with P. berghei ANKA. Disease progression, stage, and survival rates were registered over 14 days postinfection. Mouse body temperatures were evaluated at baseline and postmalaria induction (at stage III/IV or at day 14 for those that survived the observation period). Mice received the TRPV1 antagonist SB366791 (0.5 mg/kg, s.c., twice a day) or vehicle (10% DMSO in saline), from 24 h postinduction of malaria. Results represent the mean + SEM of all mice per group, obtained from three independent experiments. n is indicated on each graph. ∗ p < 0.05 differs from baseline readings; # p < 0.05 differs from infected WT mice treated with vehicle. [file 9451671.f1.pdf]

TRPV1 contributes to cerebral malaria severity and mortality by regulating brain inflammation

Domingos Magno Santos Pereira[1], Simone Aparecida Teixeira[2], Oscar Murillo[3], Erika Paula Machado Peixoto[3], Mizael Calácio de Araújo[1], Nágila Caroline Fialho Sousa[1], Valério Monteiro-Neto[1,4], João Batista Calixto[5], Thiago Mattar Cunha[6], Cláudio Romero Farias Marinho[3], Marcelo Nicolás Muscará[2], Elizabeth Soares Fernandes[1]*

[1]Programa de Pós-graduação, Universidade CEUMA, São Luís, MA, Brazil; [2]Departamento de Farmacologia, Instituto de Ciências Biomédicas, Universidade de São Paulo, São Paulo, SP, Brazil; [3]Departamento de Parasitologia, Instituto de Ciências Biomédicas, Universidade de São Paulo, São Paulo, SP, Brazil; [4]Centro de Ciências da Saúde, Universidade Federal do Maranhão, São Luís, MA, Brazil; [5]Centro de Inovação e Ensaios Pré-Clínicos-CIEnP, Florianópolis, SC, Brazil; [6]Departamento de Farmacologia, Faculdade de Medicina de Ribeirão Preto, Universidade de São Paulo, São Paulo, SP, Brazil

Running title: TRPV1 deletion attenuates cerebral malaria

[#]Address correspondence to Elizabeth S. Fernandes; elizabeth.soares@ceuma.br
Telephone number: +55 98 3214-4252

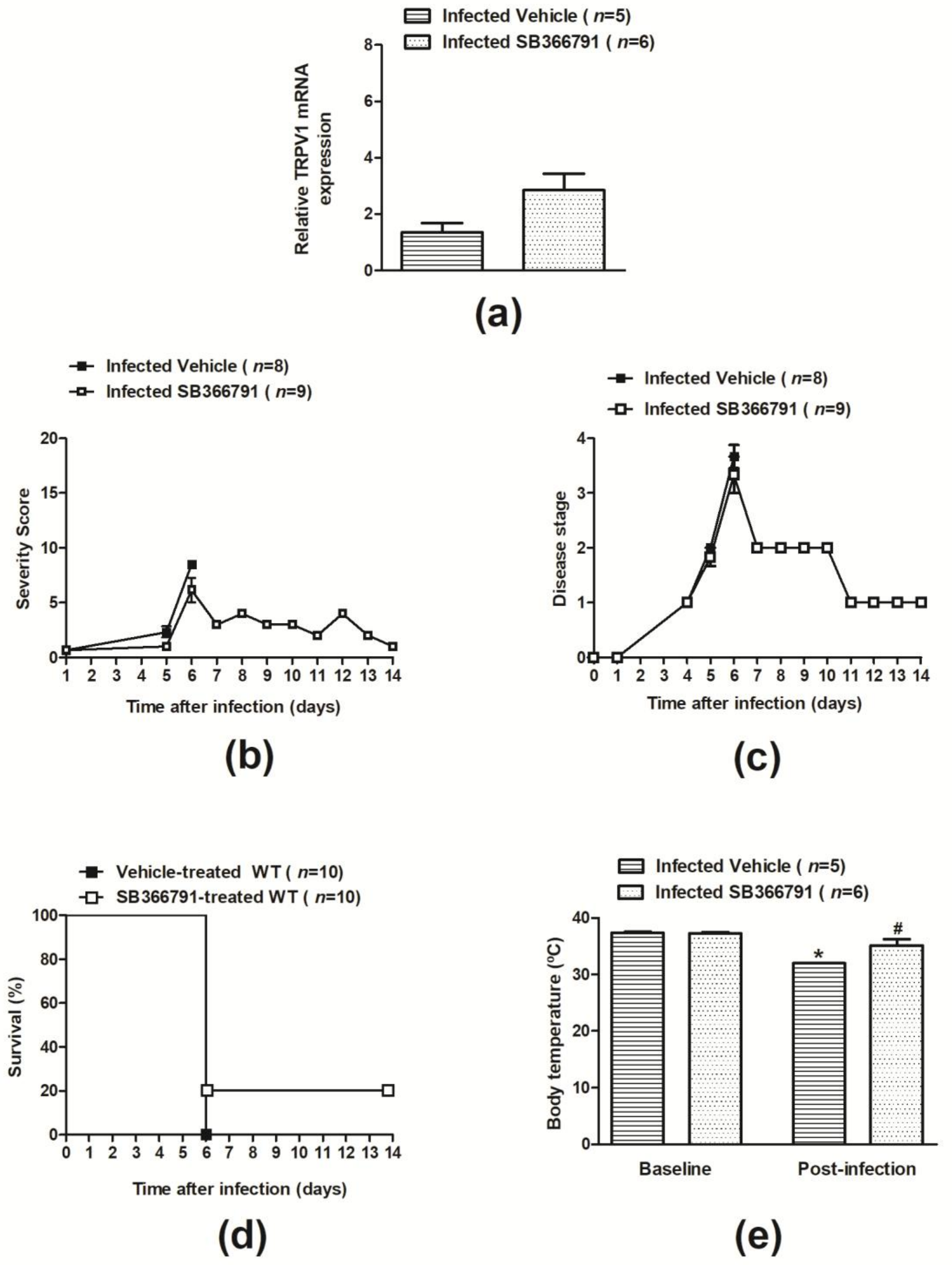

Figure S1. Effect of the selective TRPV1 antagonist SB366791 in the brain expression of TRPV1 mRNA and in cerebral malaria progression. (a) TRPV1 mRNA expression in brain samples of infected (at stage III/IV) TRPV1 wild type (WT) mice. Disease progression (b) and stage (c); survival rates (d) and body temperature (e) recordings from TRPV1 WT mice infected with *P. berghei* ANKA. Disease

progression, stage and survival rates were registered over 14-days post-infection. Mouse body temperatures were evaluated at baseline and post-malaria induction (at stage III/IV or at day 14th for those that survived the observation period). Mice received the TRPV1 antagonist SB366791 (0.5 mg/kg, s.c., twice a day) or vehicle (10% DMSO in saline), from 24h post-induction of malaria. Results represent the mean ± SEM of all mice per group, obtained from two independent experiments. *n* is indicated on each graph. Data were analysed by repeated measures analysis of variance (ANOVA) followed by the Bonferroni test with FDR correction (panels b and c). Paired and unpaired *t* test were used when appropriate (panels a and e). Survival curves were analysed by the non-parametric Mantel-Cox test (panel d). $^{*}p<0.05$, differs from baseline readings; $^{\#}p<0.05$, differs from infected WT mice treated with vehicle.
